# Solid-State Fermentation: Applications and Future Perspectives for Biostimulant and Biopesticides Production

**DOI:** 10.3390/microorganisms11061408

**Published:** 2023-05-26

**Authors:** Alessandro Mattedi, Enrico Sabbi, Beatrice Farda, Rihab Djebaili, Debasis Mitra, Claudia Ercole, Paola Cacchio, Maddalena Del Gallo, Marika Pellegrini

**Affiliations:** 1 Department of Life, Health and Environmental Sciences, University of L’Aquila, Via Vetoio, Coppito, 67100 L’Aquila, Italy; alexmtt89@gmail.com (A.M.); enrico.sabbi@student.univaq.it (E.S.); beatrice.farda@graduated.univaq.it (B.F.); rihab.djebaili@guest.univaq.it (R.D.); claudia.ercole@univaq.it (C.E.); paola.cacchio@univaq.it (P.C.); maddalena.delgallo@univaq.it (M.D.G.); 2 Department of Microbiology, Raiganj University, Raiganj 733134, India; debasismitra3@raiganjuniversity.ac.in

**Keywords:** biostimulants, biopesticides, bioactive compounds, industrial scale-up, fermentations, waste recovery, waste bioconversion, circular economy

## Abstract

With the expansion of the green products market and the worldwide policies and strategies directed toward a green revolution and ecological transition, the demand for innovative approaches is always on the rise. Among the sustainable agricultural approaches, microbial-based products are emerging over time as effective and feasible alternatives to agrochemicals. However, the production, formulation, and commercialization of some products can be challenging. Among the main challenges are the industrial production processes that ensure the quality of the product and its cost on the market. In the context of a circular economy, solid-state fermentation (SSF) might represent a smart approach to obtaining valuable products from waste and by-products. SSF enables the growth of various microorganisms on solid surfaces in the absence or near absence of free-flowing water. It is a valuable and practical method and is used in the food, pharmaceutical, energy, and chemical industries. Nevertheless, the application of this technology in the production of formulations useful in agriculture is still limited. This review summarizes the literature dealing with SSF agricultural applications and the future perspective of its use in sustainable agriculture. The survey showed good potential for SSF to produce biostimulants and biopesticides useful in agriculture.

## 1. Introduction

Sustainability perception has changed significantly since the United Nations (UN) adopted the Sustainable Development Goals (SDGs) in 2015 [1]. Through the promotion of the circular economy, the advancement of renewable energy sources, and more sustainable agriculture, global policies and strategies began social and economic fundamental changes to achieve the green revolution and ecological transition [2,3]. One of the relevant challenges in the achievement of a sustainable agrifood system is the increasing demand for biostimulants and biopesticides to limit or substitute the use of synthetic chemicals [4]. Even if many scientific questions remain unanswered, it has become more and more common to use biostimulants; these products have been extensively used in agriculture, horticulture, and forestry, to promote growth, improve nutrient uptake, protect plants from biotic and abiotic stress [5]. Their potential applications are studied to reduce our dependence on conventional fertilizers: despite their importance during the XX century to increasing crop yields for a growing population, their excess and abuse can lead to significant pollution [6]. Beyond the need for nutrients, crops are at constant exposure to hazards from parasites and other organisms that feed on them. In nature, plants defend themselves through a wide and astonishing range of mechanisms and traits [7,8], including mechanical defenses [9], structural traits [10], and particularly chemical compounds that are disgusting or toxic to phytophages and herbivores [11,12,13] or attract predators and parasitoids [14] or can contrast the attack of pathogens [15,16] or induce systemic acquired resistance which can help against future aggressions [17]. Plants have undergone millions of years of natural selection that defined their characteristics and adaptations, but human agriculture in the last ten millennia reshaped the traits of cultivated plants by intentional manipulation through the means of artificial selection, thus changing their fitness and pressures in what is considered an extraordinary example of plant-animal mutualistic co-evolution [18,19,20]. In agriculture, the study of the ecological interactions and the evolutionary patterns related to plant exposure to abiotic and biotic stresses is particularly concerning, as they can significantly reduce yields and damage crop quality, which are necessary to feed people. It is not only a matter of the farmer aiming at saving the entire harvest and avoiding any minor losses: modern cultivars are more productive, but also often more vulnerable compared to their wild progenitors because of a complex interaction of factors, including reduced genetic variability and loss of secondary toxic metabolites [21,22,23,24]. Plant phytophages and pathogens can and will lead to dire consequences regarding food provisioning and waste [25,26] economic losses [27] risk of food poisoning due to toxins [28]. The current climate emergency is also a Damocles’ sword as global warming will further spread certain phytophages and pathogens, while also exerting higher abiotic stresses on crops, thus exacerbating plant diseases and physiopathies. Third-world countries will be the most impacted, but first-world countries face risks to their food security too [29,30,31]. In the last century, scientific and technological research developed several ways to mitigate or deter these issues. One of the most important tools is agricultural chemistry. Since damaging organisms are vernacularly termed pests, chemical compounds that repel or kill them are termed pesticides. It is not surprising that pesticides have occupied a key role in agriculture in the last decades to assure a stable source of food and income for farmers, but also to fight the spread of diseases, and overall to sustain a constantly growing population with its increasing demands [32]. However, they did not come without possible risk due to their abuse and mismanagement, and in some cases even from their application alone. While the benefits of agrochemistry have been widely recognized, there was also increasing concern for the collateral effects on the environment [33,34,35] with a particular focus on human health [36] non-target organisms [37], the development of resistance in target organisms [38,39,40], and economical costs [41]. Despite this, many farmers still rely on pesticides, which can be seen as counterintuitive, and their consumption has increased worldwide as they often are necessary, particularly herbicides followed by fungicides and insecticides [42]. These issues are of no quick and easy solution. A further problem is that countries in the third world suffer the most harm from crop damage, as they are economically vulnerable, and farmers often lack the instruments and the money to adequately face the most severe issues and recover from losses. They are also more vulnerable to the effects of climate change. The Food and Agriculture Organization of the United Nations estimated that up to 40% of global crop production is lost because of pests and that the current climate change scenarios will result in an increase in pest risk and pesticide usage in agricultural ecosystems. [43]. Agricultural research was and currently is pivotal in the struggle against poverty [44]. Yet, farmers in the poorest countries cannot always afford the most effective and efficient tools, are forced to renounce chemical compounds to protect their crops, or to use older formulations that might be more polluting (sometimes banned in first world countries) and faulty, less safe instruments to release pesticides [45]. In the last decades, research has focused on how to mitigate the environmental effects of pesticides, reduce the need for them, and find alternative tools and strategies for their use. The global goal is to achieve a more sustainable agriculture, while not decreasing yields [46]. Therefore, research in plant protection is key to these ambitious objectives. This has been pursued through more severe regulations, increased technical education [47,48] and the development and choice of less impacting agrochemicals that are more selective or less persistent [49,50,51], the breeding or engineering of resistant crops [15], the application of evolutionary and ecological thought in agriculture to improve crop selection [19], the optimization of farming practices through systems such as integrated and precision agriculture and ecological intensification [52], search of microorganisms for biological control [53], the usage of useful insects for biological control [14]. The importance of research in crop protection is increased considering the impact of climate change on agriculture, and the need to reduce the impact on the climate of agriculture itself. Reducing the need for agrochemicals and increasing the efficiency of farming techniques can help reduce the emissions of carbon dioxide and greenhouse equivalents [54].

Midst new bio-based products, the use of microbial-based inoculants are gaining increasing interest from research, industrial, and commercial fields. Microbial-based inoculants contain microbial entities with the ability to increase nutrient uptake, shield plants from biotic and abiotic stress, and promote growth (e.g., germination, flowering, fruiting) [5]. These microbes fall within plant growth-promoting microbes (PGPM), beneficial bacteria and fungi that sustain the positive effects on plants by colonizing plant roots and benefit their hosts by controlling the synthesis of phytohormones, boosting soil nutrient availability, and enhancing disease resistance [55].

Since the discovery and description of PGPM, scientific research carried out the isolation and characterization of countless potentially useful strains. However, most of these isolates are not commercialized [56]. The commercialization of bio-based products may be severely hampered by improper microbial inoculant formulation that may not consider the costs linked to the industrial production of the product and its input into the market [57,58]. A successful microbe-based formulation is characterized by efficacy, versatility, practicality, delivery, persistence, commercial viability, and congruity with regulatory frameworks [57]. These aspects, which ensure the high quality of the product and its success in the market, are achieved through a valid scale-up from laboratory to industrial production. The fundamental concerns of industrial fermentations, process optimization, and scale-up are targeted at maintaining optimal and uniform reaction conditions, limiting microbial stress exposure, and boosting metabolic accuracy to maximize product yields and assure consistent product quality [59]. A thorough and detailed process characterization, the identification of the most important process parameters influencing product yield and quality, and their establishment as scale-up parameters to be kept constant as much as possible are required to develop suitable strategies for each individual product, process, and facility [59].

Among the processes applied in the production of microbe-based products (e.g., microbial biomass, enzymes, cell metabolites, etc), submerged state fermentation (SmSF)—also known as liquid state fermentation or submerged liquid fermentation—is the most used technology [60]. However, based on the requirements of microbe/microbes, the growth media, resources, and energy inputs (i.e., large amounts of water and costs of agitation and aeration), and equipment, SmSF could be expensive, resulting in a non-economically sustainable process [61]. Moreover, SmSF is sensitive to several factors, prone to contaminations, has a lack of control of the physical and chemical variables of the process, and some enzymatic and metabolite releases might be challenging [61]. Solid-state fermentation (SSF)—in which bacteria and fungi, are grown on a moist, solid, non-soluble organic material in the absence or almost absence of free-flowing water—is used as an alternative to SmSF for several microbial biotechnology processes [62]. Beyond low energy consumption and other practical advantages than SmSF, SSF allows the bioconversion of organic agricultural and industrial wastes, achieving the circular economy goal [63]. 

Huge quantities of residues are created annually by industries with agricultural backgrounds. If these leftovers aren’t properly disposed of, they might pollute the environment and have a negative impact on both human and animal health. Because most agro-industrial wastes are untreated and unused, they are often disposed of by burning, dumping, or unintentional landfilling. These untreated wastes increase several greenhouse gases, which contribute to various climate change issues [64,65]. The recent review of Yafetto emphasized the significance of using SSF to valorize diverse agro-industrial wastes to produce goods with advantages for industry, agriculture, and human health [66].

In view of the need for agro-industrial wastes valorization and economic sustainability of microbial-based products improvement, the purpose of this review is to underline the application of SSF in biostimulants and biopesticides production and encourage research to progress knowledge on the subject. A detailed description of the technology and the laboratory- and industrial-scale instruments were provided. To evaluate the suitability of SSF for this purpose we summarised the literature dealing with the topic using several databases. Limitations, advantages, and future perspectives were also presented.

## 2. Solid-State Fermentation: Process and Applications

SSF is a three-phase heterogeneous process that combines solid, liquid, and gaseous phases to convert a starting substrate to products with added value. SSF has drawn a lot of interest in the last two decades for the development of industrial bioprocesses, because of its economic and environmental sustainability whilst producing more products with a decreased risk of contamination. Several parameters affect SSF and are essential to the process development’s technical and financial viability. As with other bioprocesses (including SmSF), these parameters include the selection of the right microbe/consortium and substrate, and finding the best physical, chemical, and biological process parameters (e.g., pH, aeration, temperature, humidity, solid material characteristics). The purification of the product is an additional element that has an impact on SSF production feasibility. Throughout the fermentation heat accumulation and the heterogeneous nature of the substrate (a complex gas-liquid-solid multiphase system) are two of the main SSF problems to overcome in scale-up. Beyond the use of SSF for biopesticides and biostimulant production—discussed in detail in the following sections—SSF demonstrate to be a valid process in several fields. SSF is commonly utilized in the production of metabolites (e.g., antibiotics, aromas, biosurfactants, enzymes, organic acids) biofuels, and environmental purposes (e.g., bioremediation) [62,67]. These productions are carried out employing different types of bioreactors. The following section describes the most common types of devices and the different types of configurations from laboratory to industrial scale.

## 3. Solid-State Fermentation Bioreactors

**Figure 1:**
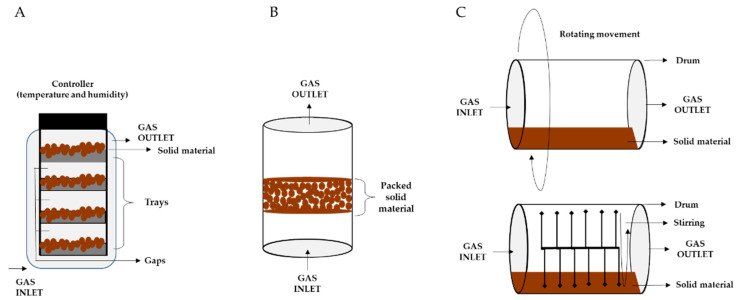
SSF bioreactors with occasional agitation and without forced aeration ((**A**) Tray bioreactor); with occasional agitation and forced aeration ((**B**) Packed-bed bioreactor); and with slow continuous agitation and without forced aeration ((**C**) Two models of stirred drum bioreactors) [69].

The laboratory-scale SSF devices consist of inert supports (e.g., Petri dishes, flasks, and bottles) that can be used to process a few grams of matrix to carry out rapid screenings (e.g., inoculum-to-matrix ratios, optimal temperature). Generally, at this scale, the temperature is the only parameter controlled and no forced aeration or agitation is applied [68]. Once reached the optimal lab scale conditions (e.g., inoculum rate, matrix quantities, optimal temperature) pilot and industrial scale processes are studied and optimized in bioreactors with sophisticated control systems. There are many types of bioreactors that mainly differ based on the presence or absence of agitation and forced aeration [69]. The simplest type is the tray bioreactor (Figure 1A) in which the solid material is laid on trays constructed with inert material (e.g., metal, wood, plastic). Trays are placed in a tray chamber with a suitable gap among them in which a circulating air controls temperature and humidity. In tray bioreactors, the air is not forced, and agitation might occur occasionally based on the process carried out [69]. 

In the presence of occasional agitation and forced aeration we can find packed-bed bioreactors (Figure 1B), glass or plastic column reactors in which the solid material is packed inside it. Aeration is guaranteed by fluxing air from the bottom and the temperature is maintained by external devices (e.g., heat exchangers or cooling/heating jackets). Packed-bed bioreactors can be used also in the presence of intermittent mixing and forced aeration, providing the agitation by a mechanical stirrer or airflow [70].

For SSF that need slow continuous agitation and no forced aeration, there are two types of stirred drum bioreactors (Figure 1C). In these types of bioreactors, the solid material is filled within the drum, and air is blown in it. The agitation is assured by a rotating drum in the rotating-drum bioreactor (above) and by paddles inside the drum unit in the stirred-drum bioreactor (below).

**Figure 2:**
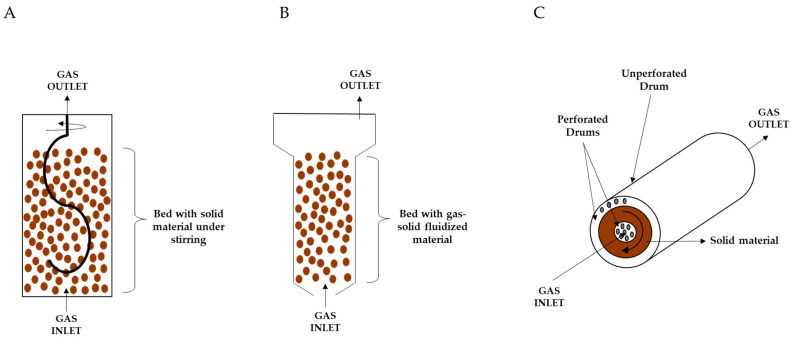
SSF bioreactors with continuous agitation and forced aeration. (**A**) Stirred aired bioreactor; (**B**) Gas-solid fluidized bed bioreactor; (**C**) Rocking drum bioreactor [69].

In SSF with slow continuous agitation and forced aeration, there are three types of bioreactors that can be used including stirred-aerated bioreactors (Figure 2A), gas-solid fluidized beds bioreactors (Figure 2B), and rocking drums bioreactors (Figure 2C). These reactors vigorously blow air through the bed while agitating it. Depending on the type of mixing, such a bioreactor can normally be operated in one of two modes: continuously mixed or intermittently mixed bioreactors. Thanks to the addition of water to the bed, the mixing system reduces the cooling demand. The sensitivity of the microorganisms to shear effects from mixing as well as the mechanical and sticky characteristics of the substrate particles will determine whether continuous or intermittent mixing should be used [68,69,70]. Although several studies used and set up a wide variety of alternatives to these fermenters, the tray- or drum-type bioreactor still serves as the starting model to design them [69].

## 4. Solid-State Fermentation for Biostimulants Production

Many works focused the investigations on the SSF application to produce biostimulant agents. Table 1 summarises the existing literature on the subject, focusing on the last 10 years. Only a few studies evaluated the suitability of SSF to produce biostimulant agents and tested the SSF products on plants. Almost the entirety of the reports focused on the study of *Trichoderma* spp., considering a wide range of substrates, and evaluating the biostimulant effects of SSF products on different horticultural and officinal plants. Among these studies on *Trichoderma* spp. noteworthy is the recent study of Liu et al., in which it has been demonstrated the suitability of rice straw in combination with other natural-derived amino acids as SSF substrate to improve the population of *T. guizhouense* NJAU4742 and develop an innovative biostimulant capable to improve pepper growth and development [71]. Biostimulant production through SSF was also valid in other studies involving other fungal and bacterial strains. Romano et al., for example, demonstrated the suitability of SSF with vermiculite, exhausted yeasts, and vinasse to produce a *Kosakonia pseudosacchari*-based biostimulant able to induce plant growth and development on maize [72]. Most of the studies on these other microbes are single reports or described by only one group of scholars. The exception is *Bacillus* spp.; for the latter, several optimized SSF processes have been used to produce spores useful for biostimulating action on various crops. Other environmental useful applications were described for *Bacillus* spores produced by SSF processes. Rodriguez-Morgado et al., for example, efficiently used *Bacillus licheniformis* spores derived from SSF of sewage sludge to improve soil biochemical characteristics (e.g., ergosterol concentration and enzymatic activity).

## 5. Solid-State Fermentation for Biopesticides Production

Biopesticides are a wide category of compounds of biological origin that can exert an antagonistic effect against other organisms, with deterring, competitive, or biocidal effects. The definition is debated in the literature, as different authors and regulatory agencies might disagree on the inclusion of certain products depending on their method of action or source [80]. The Food and Agriculture Organization defines biopesticides as “A generic term generally applied to a substance derived from nature, such as a microorganism or botanical or semiochemical, that may be formulated and applied in a manner similar to a conventional chemical pesticide and that is normally used for short-term pest control” [81]. The European Union defines pesticides or biological control products as “substances used to suppress, eradicate and prevent organisms that are considered harmful”, including both plant protection and biocidal products, and specifies that biopesticides are a subcategory “comprised of substances that are derived from living organisms and certain minerals” [82]. The Environmental Protection Agency of the United States (EPA) divides biopesticides into three types: biochemical, microbial, and plant-incorporated-protectants. 

Microbial biopesticides are the most common, and the subjects of this review. They consist of microorganisms such as bacteria, fungi, and protists, which can directly suppress target organisms, or produce compounds that have properties that are useful to control or eliminate them [83]. Certain authors include viruses too [84,85]. The rationale behind the use of biopesticides is the idea that they are generally less persistent and more degradable than traditional pesticides, but also more target-selective, thus reducing their environmental impact. They are expected to be more economical than conventional pesticides, which is the main interest for poor countries, although certain limitations discussed in the following sections can increase costs. The intended goal is to allow to effectively fight pests without significant crop losses. There is increasing scientific literature showing interest in their potential applications, their production, their enhancements, their limitations to be overcome, the technical side of their employment, and the legal and economic aspects [86,87,88,89,90,91]. The EPA particularly suggest them as a component of integrated pest management that is capable of effectively reducing the use of conventional pesticides while keeping high yields [92]. 

The scientific literature contains many reports on the use of SSF to produce biopesticides. Supplementary Table S1 (Supplementary Material) and Table 2 summarise the existing literature on the subject focused on the last 10 years, with some notable cases from the previous period. Most of the scientific papers deal with the use of SSF to grow fungi and oomycetes (Supplementary Table S1, Refs. [93,94,95,96,97,98,99,100,101,102,103,104,105,106,107,108,109,110,111,112,113,114,115,116,117,118,119,120,121,122,123,124,125,126,127,128,129,130,131,132,133,134,135,136,137,138,139,140,141,142,143,144,145,146,147,148,149,150,151,152,153,154,155,156,157,158,159,160,161,162,163,164,165,166,167,168,169,170]) and produce biopesticides targeting insects, nematodes, molds, and weeds. A wide variety of growth substrates can be employed. The most common substrates are wheat bran and straw, rice, and barley. Coffee husks, sugarcane bagasse, and sorghum, also are often used. More exotic substrates such as palm kernel cakes or forage cactus pears gave interesting results, which can be important for tropical countries. Even if cereals are the most common (e.g., wheat, rice, wheat bran), several works employed food and agricultural wastes. In the recent work of Ghoreishi et al., for example, grass clippings and pruning waste are used as substrate in SSF to grow *Trichoderma harzianum* and produce biopesticides (i.e., conidial spores) useful against phytopathogenic molds [170]. In the same work, the optimization of the growth parameters (mainly moisture and fermentation time) and ratios of the substrates (i.e., tryptophan, grass, and pruning waste) allowed the enhancement of 3-indole acetic acid and spores recovery.

As summarized in Table 2, the production of biopesticides through SSF is also possible with the use of bacterial inocula. *Bacillus* spp. are the commonly used inocula to produce biopesticides targeting phytopathogenic bacteria, molds, mosquitos, and insects using diverse substrates (including different wastes). Specific targets are *Culex pipiens*, *Rhizoctonia solani*, *Agrobacterium tumefaciens*, *Colletotrichum lini*, *Fusarium oxysporum*, and *Phytophthora palmivora*. *Bacillus thuringiensis* is by far the most successful bacterial biopesticide, due to its efficacy against insects and readily available, with strains selected for specific targets [171,172,173,174]. Most of its success depends also on the fact that it is considered safe for mammals, humans included, although it has been reported that in rare cases some strains might affect human health through enterotoxins [175]. A few studies also reported the inoculation of actinomycetes for antimicrobial agents. The literature survey also showed promising applications of bacterial strains SSFs for biopesticides production. *Rhizopus oligosporum* SSF, for example, is commonly used to produce food (tempeh) [176]. However, the same technology has not been yet applied for the optimization of potential biopesticides released by this species (e.g., antifungal chitinases) [177]. Also, the recent work of Widyastuti et al. described the *Pseudonocardia antitumoralis* 18D36-A1 SSF using shrimp shell wastes to produce antifungal biopesticides against Malassezia globose (a mammalian pathogen) [178].

**Table 2:** Literature on solid-state fermentation (SSF) use to produce biopesticides employing bacterial inocula.

Species	Tg	Growth Substrates	T (°C)	D	Maximum Biomass Yield	Tested Against	Ref.
*Bacillus amyloliquefaciens* BS20	B	feed-grade soybean meal, corn flour, and wheat bran	37 °C	2	7.24 × 10 ^10^ CFU/g	*-*	[179]
*Bacillus amyloliquefaciens* B-1895	B	corncobs	37 and 32 °C	4	47 × 10 ^10^ spores g^−1^ biomass	*-*	[180]
*Bacillus amyloliquefaciens* HM618	M	food waste “mainly included rice, vegetables, and small amounts of soup”	-	-			[181]
*Bacillus sphaericus* NRC69	Q	wheat bran, rice hull, wheat straw, corn stover, corn cobs, cotton stakes, olive meal, date stone, pea peels, potato peels	30 °C	6	116 × 10 ^9^ CFU/g	*Culex pipiens*	[182]
*Bacillus sphaericus* 14N1 *Lysinibacillus sphaericus*	Q	wheat germ meal, linen meal (4.5% each), and 0.2% yeast extract with fine sand as the carrying material	30 °C	5	-	*Culex pipiens*	[183]
*Bacillus subtilis* RB14-CS	M	soybean curd residue (okara) + some other nutrients	25 °C	5–10	2.75 × 10 ^9^ CFU/g dry soil	*Rhizoctonia solani*	[184]
*Bacillus subtilis* RB14-CS	M	soybean curd residue (okara).	25 °C	4	10 ^10^ to 10 ^11^	*Rhizoctonia solani*	[185]
*Bacillus subtilis* RB14-CS	M	soybean curd residue (okara).	25 °C	6	-	*-*	[186]
*Bacillus subtilis* SPB1	B	dried and grinded seeds of Aleppo pine	37 °C	2	27.59 ± 1.63 mg/g	*Agrobacterium tumefaciens* C58	[187]
*Bacillus thuringiensis* var *israelensis* CECT 5904	I	bulking agent was mixed with digestate and biowaste	30, 37, and 45 °C	3	4 × 10 ^8^ spores g^−1^ DM	*-*	[188]
*Bacillus thuringiensis* var *kurstaki* NRRL HD-73 (CECT 4497)	I	organic fraction of municipal solid waste	27 °C	4	10 ^9^ CFU g DM^−1^	*-*	[189]
*Bacillus thuringiensis* var *kurstaki* HD-73 (ATCC-35866)	I	polyurethane foam	30 °C	21–36 h	8 × 10^9^ mL^−1^	*-*	[190]
*Bacillus thuringiensis* var *israelensis* CECT 5904	I	OFMSW, three cosubstrates	30 °C	3	1.1 × 10^9^ spores g^−1^	*-*	[191]
*Bacillus thuringiensis* var *israelensis* CECT 5904	I	digested sewage sludge and digested OFMSW cosubstrates on the solid residue after enzymatic hydrolysis of OFMSW	39/42	4	10^8^–10^11^ spores g DM^−1^	*-*	[192]
*Pseudomonas aeruginosa* LYT-4	M	tung tree (*Vernicia fordii*)	30 °C	6	-	*Colletotrichum lini, Rhizoctonia solani* and *Fusarium oxysporum*	[193]
*Streptomyces* sp. K61	M	silica, cornsteep solids, dolomite lime, lactose	22–28 °C	5 + 5	4.5 × 10 ^9^ CFU g ^−1^	*-*	[169]
*Streptomyces gilvosporeus* Z28	M	blend of rapeseed cake, rice hull, wheat bran and crude glycerol	28 °C	10	-	*-*	[194]
*Streptomyces griseorubens* JSD-1	B	peat soil with 2% *w*/*w* rice husk	32 °C	7	1.69 × 10 ^9^ CFU g^−1^	-	[195]
*Streptomyces hygroscopicus* B04	M	various combinations of rapeseed meal, wheat bran, and vermicompost	28 °C	5–7	1.34 × 10 ^9^	*Fusarium oxysporum*	[196]
*Streptomyces similanensis* 9X166	M	rice bran, cassava chips, and coconut husks	33 ± 2 °C	7	151 × 10 ^7^ CFU/g^−1^	*Phytophthora palmivora*	[197]

In the table, Tg, Targets:B, bacteria; M, molds; I, insects; Q, mosquitos; D, SSF days; DM, dry matter.

Even if these products have a promising future, there literature survey also unveiled some issues that must be discussed. Several formulations have limited effectiveness and consistency compared to traditional pesticides (they can be slow to kill and take time to reduce pest populations), shorter shelf life both in storage and in the field (ironically a side effect of their high biodegradability), the differing standard method of preparations and guidelines, more labor-intensive application management, more difficult storage and handling, difficulty in scaling-up for large production, consequential increase in costs in refined products despite the initial cheapness of starting ingredients [87,88]. Biopesticides are considered at risk of selecting resistance in target species, mainly because of a lack of management compared to other methods, but the concern is inferior to that for conventional pesticides, particularly when dealing with biopesticides based on infection means rather than toxins. To reduce this risk, it has been proposed to use a wide array of biopesticides in a heterogeneous landscape, valorizing diversity to reduce chances for selection [198]. There are some reports about possible underrecognized out-of-target toxicity of biopesticide formulations, that require further attention, management, and investigation [199,200,201]. Another recent source of concern is that certain microbial biopesticides could be reservoirs of antibiotic resistance, but this field is still mostly unknown and under investigation [202]. Lack of information and awareness can also limit the diffusion of biopesticides in third-world countries [203]. Recent reports unveiled that several Indian microbial biopesticide-based solutions have quality problems (e.g., impurities, the excessive moisture content in solid formulations, or fewer colony propagules than stated). More than 50% of these products do not fulfill Central Insecticides Board and Registration Committee (CIBRC) standards [204]. However, as the demand for more sustainable and environmentally friendly pest management solutions continues to grow, research into new and improved biopesticides is likely to increase, and several formulations are already currently commercialized, standards of production are being defined, and new regulations are being approved. The current studies investigating their applications and reviewing their benefits, limits, and possible developments, are already showing that research is increasing [205,206,207,208,209,210,211]. Their main application will be within integrated pest management strategies [210]. There are also proposals for genetically engineering biopesticides [211,212,213].

## 6. SSF for Sustainable Agriculture: Advantages and Limitations

As presented in the previous section, SSF has been used to cultivate a wide range of microorganisms because it offers several potential advantages. SSF mimics the natural condition of growth for many microorganisms. It is considered cheap and of low impact, as it can rely on easily available substrates such as food discards, agriculture wastes, and urban wastes. Cost productions are one of the major limiting factors for biopesticides. Furthermore, SSF can be a way of recycling such wastes, as they are a source of pollution that must be disposed of. Another factor that could reduce costs is the fact that it does not require to use energy to heat the process [66,214,215,216]. As with other techniques, SSF has limitations. The first significant SSF constraint is directly related to heating. Fermentation depends heavily on temperature because many microbes need specific temperatures to develop, while the process itself heats things up. Since solid substrates have a low heat conductivity, air convection is the most common method of heat dissipation; however, this results in increased moisture loss and substrate drying, both of which have an impact on fermentation. Alternately, water can be added to reduce heat or drying, but this requires a mixing device that is unsuitable for the cultivation of filamentous fungi and may release nutrients that contaminants could use. Some studies have been directed towards optimizing the parameters to avoid heat accumulation. The study by Figueroa-Montero et al., for example, used the combination of mathematical models with internal air circulation by forced convection to modify the transfer of heat and water and to allow dissipation of the heat generated in the bioprocess [217]. Several other studies unveiled how matrix porosity should be studied to optimize air penetration, heat transfer, and effective air diffusion and decrease the heterogeneous nature of the substrate’s negative effects on the bioprocess [218].

Product recovery is SSF’s second significant drawback. Sometimes the finished product can be utilized right away, but metabolites frequently diffuse through solid substrates and need to be extracted, typically using large amounts of organic solvents, which greatly raises expenses and negates the initial cost savings. The substance that has been used up becomes waste that must be disposed of. When extracting metabolites, purification is a problem as well because it can be difficult and expensive. The product isolation and purification procedure depend on the product type, (e.g., intracellular or extracellular cell metabolite or whole cell biomass). Extracellular products are extracted directly from the fermented solid substrate, while intracellular products are extracted after the cell wall rapture (e.g., high-pressure homogenizer) [219]. Nevertheless, SSF uses a low water content to produce a higher concentration of products than SmSF [62]. This allows SSF to be a more cost-effective and lower solvents usage process than SmSF. The SSF constraints, the numerous approaches being used to solve them, and the potential future directions for further developing this biotechnology to increase its competitiveness in the worldwide market were all covered in detail by Oiza et al. [216].

Some other limitations can be linked to the lack of reports describing the growth behavior of some microbes. As underlined in the previous sections, the main microbes that are cultivated through SSF are filamentous fungi. They are particularly suited for SSF as they naturally colonize and decompose organic residuals and wastes. They are also competing against most contaminants; thus, it is usually not necessary to use aseptic bioreactors. They also do not require high amounts of water, their spores are particularly resistant to hostile environmental conditions, and the fermentation process nets high productivity and the final concentration of stable products. These factors render SSF more advantageous compared to SmSF for fungi. The most common issue is scaling up, as many processes are effective only on a laboratory scale and not for mass production [94,220,221,222]. Conidia produced using solid substrate fermentation are often more stable and resistant to stresses caused by drying than those produced in liquid culture [221]. No reports on the SSF cultivation of microorganisms for biopesticides of the domain of Archaea can be found. Nevertheless, these microbes are useful to produce enzymes, degrade agricultural wastes, and production of compounds valuable in food preservation and medical fields [223,224,225,226,227,228].

## 7. Conclusions and Future Perspectives

Diverse microorganisms can grow on solid surfaces without or almost without free-flowing water thanks to SSF. The food, pharmaceutical, energy, and chemical sectors all use this useful process. However, this technology’s use in creating formulations useful for agriculture is still in its infancy and several key issues need to be addressed. The literature survey revealed that there are several substrates that have been tested for use in SSF. Many of them can also be useful in tropical countries, where certain local agriculture products leave wastes that could be exploited. But the most common and promising substrates are the global-wide cereals, since they are among the most cultivated crops in the world, and their biochemical profile is composed of easily available nutrients for the growth of microorganisms. This can pose a supplying problem when we consider that, as a staple food, cereals are first and foremost destined for the human diet, in a similar issue to biofuel crops. Therefore, research should improve production sustainability, focusing on recycling food residuals or agro-industrial wastes. Recent literature also shows that SSF has a good potential for producing biostimulants and biopesticides that are beneficial to agriculture, but there is still much work to do to solve the current operational limitations: mainly decreasing the costs of extraction, increasing the shelf-life of the final products, increasing the final returns in cell or spore production. Improving yields can be done either by increasing productivity for a single area or by allocating more land to crop fields. Surface intensification can consume soil, but land use is a major problem for the reduction of ecosystems and biodiversity. Therefore, the challenge of the XXI century will be set in the balance between these different requirements.

New tools for plant stimulation and protection, to produce more while consuming less soil and resources, are a welcome path to pursue. Biostimulants are the subject of intense research to mitigate the excess of nitrogen in soil and water systems and reduce our dependence on fertilizers, which are needed to sustain crop yields, yet their abuse is environmentally polluting. Biopesticides currently are mainly directed against insects and molds, which are sources of major interest in agriculture, both to contrast deleterious pests and to avoid damaging useful organisms that could offer many ecosystem services like pollination, predation of phytophages, biomonitoring, etc. Bioinsecticides, as evidenced in our search, are important for sanitary reasons too, to contain mosquitoes which are vectors of dangerous diseases such as malaria, dengue, or zika. Agroecosystems are sensible to the spread of mosquitoes, as they reproduce in water sources that are easily and plenty available in farmlands, from irrigation systems to stagnations, and particularly where rice is cultivated. In third world countries, dichlorodiphenyltrichloroethane (DDT) is still allowed and used as the most effective compound against mosquitoes to counter their spread, but it is environmentally persistent. The development of alternative larvicidal formulations can be helpful in reducing the need for DDT where terrible diseases are endemic. Categories like bioherbicides instead are lacking, despite SSF being a suitable method to cultivate fungi with herbicidal properties. Thus, it is necessary more research on practical and cheap strains to develop competitive products to reduce the usage of the more effective and widespread conventional herbicides.

As far as we know there is also currently no available commercial product based on protists and Archaea for the biostimulant and biopesticide markets. These overlooked lineages can be further studied for the possibility of discovering species with potentially useful capabilities and developing biotechnological processes and products that exploit them. This applied field that we discussed will therefore rely also on basic research, whose importance is evident from the fact that the world of microorganisms is still largely unknown and in the process of being discovered in its taxonomical, genetic, enzymatic, metabolic, and symbiotic components. The discovery of new microbial species or strains that could be potentially useful will play a pivotal role in the development of new formulations. Given the good number of reports that demonstrated their suitability, filamentous fungi (mainly *Trichoderma* spp.) and *Bacillus* spp. are the most promising inoculants for biostimulant and biopesticide SSF production. Considering the growing interest in bio-based products, the need for more sustainable agricultural practices, and the SSF potential for agricultural applications, the subject is worth to be investigated. Still more research is needed.

## Figures and Tables

**Table 1:** Literature on solid-state fermentation (SSF) use to produce biostimulant agents.

Species	Substrate	T (°C)	Days	Maximum Biomass Yield	Plants Promoted	Ref.
*Trichoderma* spp.	agricultural digestate	26 °C	6	689.80 ± 80.53 mg mycelium/g substrate	cress	[73]
*Trichoderma* spp.	apple, banana, and grapefruits wastes	26 °C	6	689.8 ± 80.5 mg/g substrate	cress and tomato	[74]
*Purpureocillium lilacinum*	hair waste	28 °C	8	-	tomato	[75]
*Trichoderma atroviride* strain MUCL45632	wheat bran	-	-	-	melon, pepper, tomato, and zucchini	[76]
*Aspergillus flavipes*	soybean (most suitable)	-	-	-	*Eucalyptus* clone IPB2	[77]
*Trichoderma guizhouense* NJAU4742	rice straw + amino acids	28 °C	7	4.62 × 10 ^10^ conidia	pepper	[71]
*Fusarium redolens* KY992586 (RF1), *Phialemoniopsis cornearis* MK408657 (SF1), and *Macrophomina pseudophaseolina* MF351729 (SF2)	wheat bran	28 °C	10	38 × 10 ^12^ (RF1), 14 × 10 ^11^ (SF1), and 21 × 10 ^12^ (SF2) CFU g^−1^	*Coleus forskohlii*	[78]
*Kosakonia pseudosacchari TL13*	vermiculite, exausted yeasts and vinasse	15 °C	30	7–6.9 log CFU g^−1^ or mL^−1^	maize	[72]
*Trichoderma asperellum*	silica-rich spent mushroom	28 °C	31	12.37 × 10^13^ cfu/g bioformulation	tomato	[61]
*Bacillus circulans* Xue-113168	food waste and feldspar	30 °C	7	8–10 CFU g^−1^	rapeseed	[79]

## Data Availability

Not applicable.

## Supplementary Materials

The following supporting information can be downloaded at: https://www.mdpi.com/article/10.3390/microorganisms11061408/s1, Table S1: Literature on solid-state fermentation (SSF) used to produce biopesticides employing inocula of fungi and oomycetes.
